# Life History Trade-Offs and Behavioral Sensitivity to Testosterone: An Experimental Test When Female Aggression and Maternal Care Co-Occur

**DOI:** 10.1371/journal.pone.0054120

**Published:** 2013-01-14

**Authors:** Kimberly A. Rosvall

**Affiliations:** Department of Biology, Indiana University, Bloomington, Indiana, United States of America; University of Jyväskylä, Finland

## Abstract

Research on male animals suggests that the hormone testosterone plays a central role in mediating the trade-off between mating effort and parental effort. However, the direct links between testosterone, intrasexual aggression and parental care are remarkably mixed across species. Previous attempts to reconcile these patterns suggest that selection favors behavioral insensitivity to testosterone when paternal care is essential to reproductive success and when breeding seasons are especially short. Females also secrete testosterone, though the degree to which similar testosterone-mediated trade-offs occur in females is much less clear. Here, I ask whether testosterone mediates trade-offs between aggression and incubation in females, and whether patterns of female sensitivity to testosterone relate to female life history, as is often the case in males. I experimentally elevated testosterone in free-living, incubating female tree swallows (*Tachycineta bicolor*), a songbird with a short breeding season during which female incubation and intrasexual aggression are both essential to female reproductive success. Testosterone-treated females showed significantly elevated aggression, reduced incubation temperatures, and reduced hatching success, relative to controls. Thus, prolonged testosterone elevation during incubation was detrimental to reproductive success, but females nonetheless showed behavioral sensitivity to testosterone. These findings suggest that the relative importance of both mating effort and parental effort may be central to understanding patterns of behavioral sensitivity in both sexes.

## Introduction

Among the most widespread behavioral trade-offs are those between mating effort and parental effort, where individuals divide limited resources between attracting mates or repelling rivals on the one hand, and caring for offspring on the other [1]–[3]. Naturally or experimentally elevated testosterone is often associated with greater investment in mating effort via ornaments, armaments, or aggressive behavior (i.e. traits and behaviors that attract a potential mate or deter same-sex competitors), whereas lower androgens are often associated with greater investment in parental care [4]–[12]. Thus, the androgen testosterone (T) is thought to be one of the proximate mediators of this trade-off, as T can either directly (e.g. by binding androgen receptors) or indirectly (e.g. by interfering with prolactin signaling) affect the expression of aggressive and parental behaviors [13], [14].

The vast majority of research on the role of T in the trade-off between parental care and intrasexual aggression has been undertaken in males. Males with experimentally elevated T often exhibit more frequent or intense aggression towards rivals, while provisioning and/or incubating relatively less than control males [15]–[25]. However, there are many studies that do not support this pattern [26]–[29]. Attempts to reconcile these mixed results in males have focused on two main hypotheses, both of which relate to paternal care and the fact that shifts from high to low testosterone are somewhat limited in their temporal flexibility [4], [29], [30]. The ‘essential paternal care’ hypothesis suggests that if male care is especially important for reproductive success, then selection may favor behavioral insensitivity to T [29], [31], [32], and so, T should be less likely to affect behavior in species with significant paternal care. The ‘short season’ hypothesis posits that behavioral insensitivity to T should evolve when breeding seasons are especially short (e.g. in single brooded or arctic dwelling species) because selection ought to favor a rapid transition from mate attraction to parental care [27], [30], [33]–[35]. Each of these hypotheses attempts to explain cases of behavioral insensitivity to T, with the assumption that sensitivity to T should be favored when T-mediated mating effort (e.g. intrasexual aggression, mate guarding, etc.) is particularly important for reproductive success [4], [10], [27], [36] because T may enhance the ability to obtain a mate or keep away rivals (e.g. [37]). Conflict arises, of course, when components of mating effort and parental effort overlap in time, with the prediction that one or another behavior might evolve behavioral insensitivity to T.

Historically, the sexes were placed at opposite ends of the spectrum for this trade-off, with males investing primarily in mating effort and females investing primarily in parental effort [38], [39]. More recent research shows that females also display exaggerated traits and behaviors that may be important for mating effort (i.e. in attracting or acquiring mates or territories [40]–[43]), but we know much less about trade-offs between aggression and parental care and the degree to which these trade-offs are also mediated by T in females. Many of the phenotypic effects of androgens are not sex-specific (e.g. muscle development, immune function), and organizational effects of T often masculinize female phenotype [5], [44]. Furthermore, females secrete physiologically and behaviorally relevant levels of T [31], [32], and they express androgen receptors in a variety of neural and peripheral tissues [44], indicating that activational effects of T on female phenotype may extend beyond this hormone’s biochemical role in estrogen synthesis.

When applied to females, T implants have, in some cases, lead to an increase in female aggression at the expense of maternal care [45]–[48], but once again, there are exceptions to this pattern [31], [47], [49]–[51]. A full investigation of how T mediates behavioral trade-offs must also ask whether the T-mediated trade-offs seen in males operate similarly in females. This knowledge will provide insight into whether the hypotheses posed to explain male patterns of behavioral sensitivity/insensitivity can be extended to females. Furthermore, any comprehensive theory of the evolution of behavioral mechanisms must recognize that interspecific patterns of behavioral sensitivity to hormones may not simply reflect *male* life history. These patterns also may relate to trade-offs between parental effort and mating effort *in females*, as well as the degree to which there is sexual conflict in the resolution of these trade-offs in the two sexes.

As a first step towards this more comprehensive view, I measured the behavioral effects of experimentally elevated T in female tree swallows (*Tachycineta bicolor*). Specifically, I examined the hormonal underpinnings of a trade-off between female aggression and incubation behavior to ask whether hypotheses developed for explaining patterns of behavioral sensitivity to T in males also may apply to females. Tree swallows are ideal for examining this question because previous work suggests that females are faced with trade-offs between mating effort and parental effort during their relatively short breeding season. For example, focal observations suggest that free-living females trade-off investment in aggression and parental care (provisioning), and cross-fostering demonstrates that aggressive females tend to have poor quality offspring, likely due to a combination of reduced pre- and post-hatch maternal care, i.e. provisioning and incubation [52], [53]. Tree swallows are single-brooded and the time from clutch initiation to completion of breeding is ∼1.5 months [54]. Thus, while not as short as arctic breeding seasons for which the short season hypothesis was developed in male birds (e.g. ∼1 mo. [55], [56]), the tree swallow breeding season is shorter than other songbirds breeding in the same area [57]. Female care of offspring is essential to reproductive success in this species because a reduction in provisioning, incubating or brooding significantly decreases the quality or quantity of offspring produced [53], [58]–[60]. Whereas both sexes provision offspring, females alone incubate eggs [54], and thus, studying the effects of T on incubation avoids the potential confounds of male care.

Aggressive behavior in the context of competition for nesting cavities is also an essential component of reproductive success for female tree swallows, as females aggressively defend a nesting territory that includes the cavity and the several meters surrounding it. Further, evidence to date suggests that female aggressiveness indeed functions in the context of maintaining access to these territories or mates (i.e. mating effort), much like it does in males of many species. For example, female intruders are a common challenge, with most populations having an excess of one year-old female floaters that are capable of breeding but do not breed because they lack a nesting cavity [61]. Throughout the breeding season, these female floaters engage in frequent intrusions with cavity holders, and these interactions can escalate to overt aggression, with the possibility of eviction or even death [62], [63]. Females that are more aggressive are more likely to obtain a nesting cavity when cavities are limited, suggesting that selection favors females that are more aggressive during female-female competition [64]. Because females cannot mate or breed without a nesting cavity [54] and males do not respond aggressively to young female intruders [65], it may be especially important that females aggressively defend their nesting cavities against rival females in order to have reproductive success, much like male-male aggressiveness is a key component of male reproductive success by facilitating access to territories and mates.

If the short breeding season or importance of parental care shape female responsiveness to testosterone, then incubating female tree swallows should not alter their incubation behavior or their aggressive behavior in response to a T-implant. On the other hand, if the relative importance of aggression favors behavioral sensitivity to T in spite of its potential negative effects on maternal care, then T-implanted females should be significantly more aggressive and incubate significantly less than their control counterparts. Finally, sensitivity to T in one behavior but not another would instead suggest some degree of modularity of behavior, where selection can decouple one or another behavior from circulating T levels, according to the most adaptive combination of phenotypes [11], [66].

## Materials and Methods

### Ethics Statement

All research was approved by IACUC at both Duke University (A050-07-02; author’s affiliation at the time of field work) and University of Pittsburgh (0704588A) in accordance with Federal banding and collecting permit (21523-H), PA State Banding permit (BBN00227), and PA State Game Commission Special Permit for Scientific Study.

### General Methods

This study was conducted using free-living tree swallows breeding near the University of Pittsburgh’s Pymatuning Lab of Ecology in Linesville, Pennsylvania, USA (41°40′ N, 80°26′ W) in spring 2008. All swallows were breeding at nestboxes located at the Linesville State Fish Hatchery or the nearby Pennsylvania State Gamelands, where average distance (± se) between nestboxes was 114±8 m. Beginning early in the breeding season, nests were checked at least every third day to determine clutch initiation date, clutch size, and the onset of incubation. I followed each nest until hatching or failure. Females were banded with one U.S. Fish and Wildlife metal band and one color band. In addition, each female was marked with dabs of non-toxic acrylic paint on the wings and rump, to facilitate individual identification during behavioral trials [67]. This study focused on adult females (≥2 years old) to minimize potential age-related variation in female reproductive behavior because adult and subadult females may differ in many aspects of condition and reproductive behavior, including incubation [54], [68]–[70], though not aggressiveness [64]. Subadult and adult females are readily distinguished based on plumage coloration, with adult females displaying primarily greenish-blue plumage and subadults displaying brown plumage [71].

### Hormone Manipulation and Analyses

I implanted 14 control females and 14 testosterone-treated females. All females were captured using nestbox traps during mid to late incubation (mean ± se = 8.1±0.1 days after clutch completion, range: 6 to 10 days). Upon capture, a blood sample (approximately 50 to 100 uL) was collected from the alar vein into heparinized capillary tubes to assess pre-implant levels of circulating T for control and experimental females. All blood samples were collected within 5–11 min (mean ± se = 8.0±0.4 min) of capture of the bird. There was no relationship between collection time and T levels in either treatment group for pre- and post-implant samples (Pearson’s |r| <0.4, p>0.17), and so I did not control for time in further analyses. After brief treatment with a topical anesthetic, all females were given a subcutaneous implant (8 mm long) made of silastic tubing (I.D. = 1.47 mm, O.D. = 1.96 mm) and sealed at both ends with silastic glue. The implant was inserted under the skin along the flank, using a trocar needle. Experimental females’ implants were previously packed with 5 mm of crystalline testosterone, whereas control females received empty implants. Handling times during the implantation procedure were short and did not differ between treatment groups (control: 12.9±0.4 min; experimental: 12.7±0.4 min; unpaired t-test, t = −0.31 p = 0.76). Most females resumed normal breeding activities within a few hours (2.54±0.37 h, range: 0.45 to 8.75 h, as indicated by nest temperature data), though two females did not return after implantation (one control, one experimental), and these females were eliminated from further analyses, leaving n = 13 per treatment group. The latency to first incubation did not differ between the two groups (control: 2.5±0.7 hours; experimental: 2.4±0.5 hours; unpaired t-test: t = 0.16, p = 0.88). At the end of the study, females were re-captured using nestbox traps and mist-nets to remove implants (13.6±1.3 days after implantation, range: 5 to 31 d). At this time, a second blood sample was collected to confirm that the circulating T levels were significantly higher after implantation in the T-treated group than in the control or pre-implant samples, though I was unable to capture one female from each group. In addition, sufficient plasma volume was not collected from all females, thus sample sizes for hormone analyses are n = 4 for pre-implant T-females, n = 9 for pre-implant control females, n = 10 for post-implant T-females, and n = 8 for post-implant control females.

Blood samples were stored on ice in the field and centrifuged the same day. Plasma was drawn off the top with a Hamilton syringe and then stored at −20°C until hormone analyses. I quantified plasma T using a commercially available enzyme immunoassay (Assay Designs, Inc. 901-065) that has already been validated for use in female songbirds with comparable circulating levels of testosterone [49]. This assay reports high linearity (slope = 0.975, R^2^ = 0.999) and low cross-reactivity with androstenedione (7.2%) and other steroid hormones (all <1%). The assay sensitivity is 0.00567 ng/mL. Briefly, a trace amount of radio-labeled T was added to each sample, and plasma was extracted twice with diethyl ether. Each sample was run in duplicate on the plate, and T concentrations were based upon a 9-point logistic standard curve, with the help of a curve-fitting program (Microplate Manager, Bio-Rad Laboratories). To obtain final T concentrations, I corrected for sample volume and used extraction efficiencies to account for incomplete recoveries (efficiency: 93.7±0.6%). All samples were run on one plate, with intra-assay variability of 9.78%. Three samples were undetectable by the assay (n = 2 from the pre-testosterone group, n = 1 from the post-control group), and so I used the assay sensitivity (0.00567 ng/mL) to calculate a maximum possible T value for these samples, corrected for plasma volume (i.e. assay sensitivity divided by the plasma volume).

### Behavioral Measures

All 26 females were assayed for their level of aggressiveness using an established behavioral bioassay that is highly repeatable among females, independent of the female’s mate [52] and independent of the identity of the decoy [64]. Importantly, aggression scores from this simulated territorial intrusion predict a female’s ability to obtain a nesting cavity when cavities are limited [64], demonstrating that this assay maps onto an important component of reproductive competition. The assay measures the aggressive response of a focal female to a live, caged same-sex conspecific placed 1.5 m from the nestbox. For 5 min, I recorded aggressive behaviors directed at the decoy female: swoops and dives (to within 0.75 m), perching atop, hovering over or landing on the cage, and attempted pecking at the decoy. Swoops, dives, and close proximity are characteristic of natural female-female aggressive encounters, which involve aerial chases that can escalate to grappling and direct contact with one another [54]. Each female was assigned an aggression score, measured as the sum of all 5-sec intervals during which she responded aggressively to the simulated intrusion (ranging from 0 to 60 intervals). All decoy females were captured from at least 1 km away on the day of the trial and returned to their nesting site immediately afterwards, where they resumed normal breeding activities. Decoy females were not used in this study in any other way. The identity of the decoy did not affect the focal bird’s aggressiveness (n = 5 decoys used in 26 aggression trials, F = 0.66, R^2^_adj_ = −0.05, p = 0.63), and so I did not control for decoy identity in subsequent analyses. All aggression trials occurred between 0600 and 1100 Eastern Daylight Time at least two days after females were implanted (5.5±0.5 days; range: 2 to 15 days), and all trials occurred during the incubation phase of the nesting cycle.

**Figure 1:**
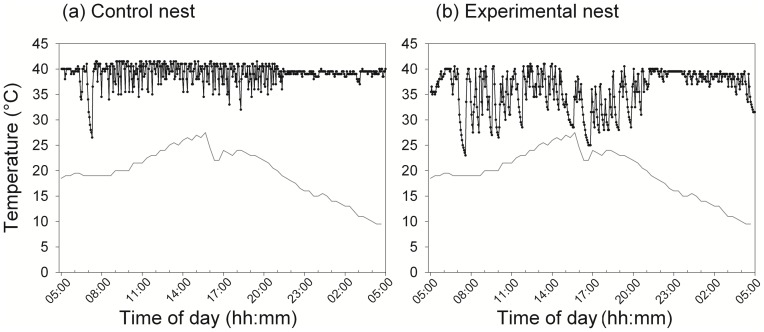
Daily temperature data. Exemplars of one full day (24 hours) of temperature data in (a) control and (b) experimental nests, beginning just before first light. Drops in temperature correspond to the female leaving the nest between bouts of incubation. Ambient temperature shown in gray line. Control and experimental nests were paired for location and date.

I quantified incubation behavior using small (17 mm diameter) battery-operated temperature sensors (iButton #1921G, Thermochron, Dallas, TX, USA). Before placing these sensors in the nest, each sensor was tested for accuracy. Only those sensors that measured within 1°C of each other were used in this study. Each iButton was then attached to a shirt button using Velcro and then wired securely into the nest cup, adjacent to the eggs. iButtons were placed in the nest on the day the female laid either the 4^th^ or 5^th^ egg of the clutch. Temperature logging began immediately after each focal bird was implanted, and the loggers recorded temperature to the nearest 0.5°C every 4th min. This technique provides a record of the temperature of the nest experienced by the eggs, and it accurately reflects the incubation behavior of females [69]. When females leave the nest, the temperature drops, and the temperature returns back to approximately 40°C when females return (**Figure 1**). In a few nests, temperature clearly tracked ambient temperature, suggesting that either the button was not placed close enough to the eggs to be incubated by the female, or that these females did not incubate at all (n = 1 control, 2 experimental). To conservatively measure temperature, I did not include these nests in future temperature analyses, although all findings are qualitatively similar if these nests are included.

I used two additional iButtons to record ambient temperature at 20 min intervals throughout the study. These loggers were placed in the shade at a central location at each of the two nearby field sites. Furthermore, treatment groups were counterbalanced by date and location to account for the possible effects of ambient temperature on nest temperatures (e.g. due to a cold snap). As a result, I recorded nest temperatures in both experimental and control nests at the same exact times on the same days. Each day included either two or four focal females, half from each treatment group. Two exceptions arose, where an experimental female stopped incubating altogether, leaving 3 nests that were observed on those dates (i.e. 2 control, 1 experimental). Thus, sample sizes for incubation analyses are n = 9 T-females and n = 12 control females.

### Statistical Analyses

**Figure 2:**
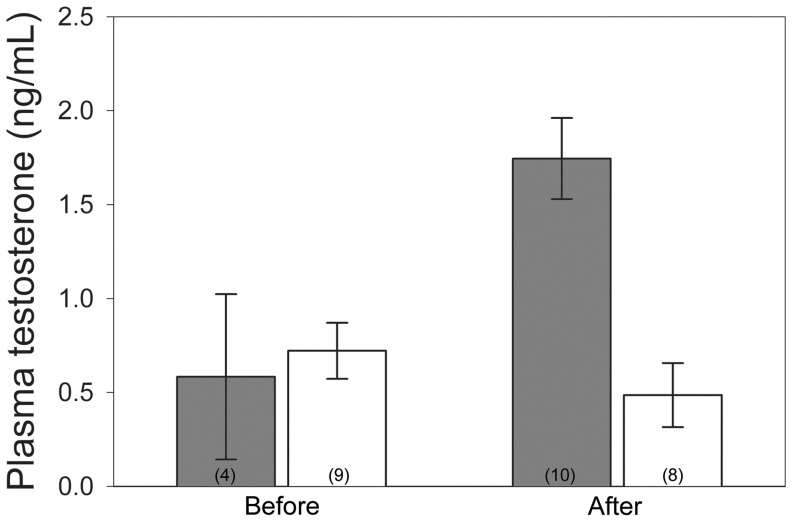
Plasma Testosterone. Experimental females (shaded bars) had significantly higher testosterone after implantation than before implantation. Experimental females also had significantly higher testosterone levels than control females (open bars). Sample sizes are shown in parentheses. These data are back-transformed from the natural log of plasma testosterone; error bars represent the standard error of these back-transformed means.

Statistical analyses were performed in JMP v. 10.0.0 (SAS Institute Inc. Cary, NC). All tests were two-tailed, and I report mean ± se. Plasma testosterone levels were natural log transformed to achieve normality for statistical analyses, and back-transformed for visual representation in Figure 2. I analyzed the effects of hormone implants using a linear mixed model (LMM) with ‘bird’ as a random repeated factor and treatment (control vs. testosterone), time (pre- vs. post-implant), and a time*treatment interaction as fixed effects.

Aggression scores were not normally distributed, and so I compared aggressiveness of control and experimental females using a Mann-Whitney U test. I compared hatching success between control and T-implanted groups with a Fisher’s exact test.

I used a general linear model (GLM) for temperature analyses, with average daytime nest temperature on the second day after implantation as the dependent variable and ambient daytime temperature and treatment as fixed effects. I selected day 2 post-implant because it allowed sufficient time for females to recover behaviorally and physiologically from capture, while still allowing all nests to be monitored a standardized number of days after implantation and before hatching (see also sensitivity analyses below).

Because hormonal and behavioral data were not collected on the same day, I performed three statistical analyses to test whether any effects of the hormone implants were likely to have been related to a short-term spike in T that had short-term effects on behavior (i.e. whether the day of sampling affected the results). Ideally, this sort of sensitivity analysis would include repeated sampling of hormones and behavior [72], though this is usually not possible in free-living animals. This study has continuous sampling of incubation behavior, and so I first asked whether T-induced changes in incubation behavior changed over time (i.e. whether incubation behavior was less and less affected by the treatment on Day 1, Day 2, and Day 4 post-implantation) using a linear mixed model with day of sampling, treatment, and day*treatment interaction as fixed effects and ‘bird’ as a repeated random factor. I also used post-hoc Tukey’s HSD tests to contrast successive days within each treatment group. Next, I used Pearson correlations to ask whether a given female’s aggressiveness was negatively related to the time elapsed since implantation with T. Finally, I asked whether T titers in T-implanted females were correlated with the number of days elapsed since implantation.

## Results

Linear mixed models on hormone data revealed a significant main effect of treatment (F_1,16.7_ = 4.64, p = 0.046) and a non-significant effect of time (pre- versus post-implant) in the whole dataset (F_1,20.0_ = 1.78, p = 0.20). However, there was a significant treatment*time interaction (F_1,20.0_ = 6.51, p = 0.019), demonstrating that T increased between pre- and post-implant samples in experimental females, but T slightly decreased over time in control females (Back-transformed means ± se: Before, control: 0.72±0.15 ng/mL, n = 9, Before, experimental: 0.58±0.44 ng/mL, n = 4; After, control: 0.49±0.17 ng/mL, n = 8, After, experimental: 1.75±0.22, n = 10, **Figure 2**). Thus, despite limited sample sizes at some time points, these data indicate the hormone implants increased plasma testosterone in the experimental group only. The mean elevated T levels in the experimental group lie at the high end of the natural range for incubating female tree swallows, reported by [73] as mean = 0.86 ng/mL, SD = 1.2 ng/mL.

**Figure 3:**
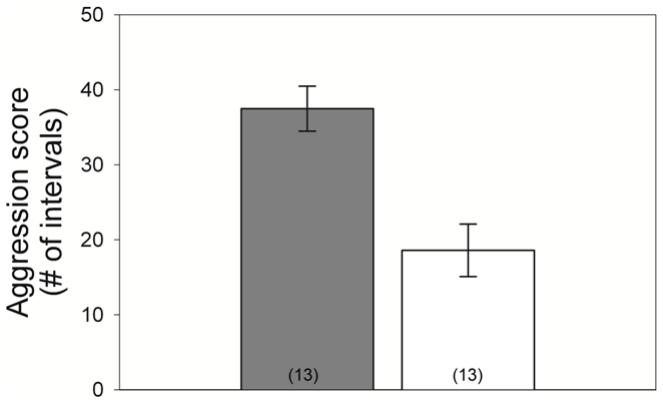
Aggression. Testosterone-implanted females (shaded bar) were significantly more aggressive than control females (open bar). Sample sizes are shown in parentheses; error bars represent the standard error of the mean.

**Figure 4:**
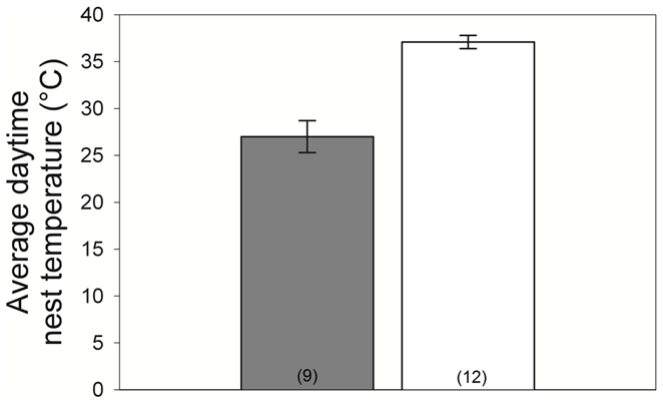
Incubation. Testosterone-implanted females’ nests (shaded bar) were significantly cooler than control females’ nests (open bar). Sample sizes are shown in parentheses; error bars represent the standard error of the mean.

Experimental females were significantly more aggressive than control females (Mann-Whitney: Z = 3.21, experimental: 37.5±3.0, control: 18.6±3.5, n = 13/group, p = 0.0012, **Figure 3**). The nests of experimental females were significantly cooler than controls, and this treatment effect was significant despite a significant effect of ambient temperature on nest temperature (GLM: χ^2^_2,18_ = 34.4, p<0.0001, ambient temperature: χ^2^ = 12.0, p = 0.0005, treatment: χ^2^ = 31.5, p<0.0001; **Figure 4**). Hatching success was dramatically different between control and experimental nests (Fisher’s exact: χ^2^ = 13.8, p = 0.0005, with 0/13 experimental nests hatching and 9/13 control nests hatching).

Temporal variation in T levels, aggression scores, and incubation temperatures suggest that these results are unlikely to have been caused by a short-term spike in T that steadily declined throughout the duration of the experiment. Among T-implanted females, there was no detectable correlation between plasma T levels and the number of days elapsed since implantation (Pearson’s r = −0.27, n = 10, p = 0.44), and there was a nearly significant positive correlation between female aggression score and the number of days elapsed since implantation (Pearson’s r = 0.55, n = 13, p = 0.052), suggesting that, if anything, the behavioral effect of the T implant increased over time, rather than decreased. LMM revealed a significant treatment effect on nest temperature (F_1,48.4_ = 13.6, p = 0.0006), a significant effect of day (F_2,37.5_ = 6.6, p = 0.0035), but no significant interaction between treatment and day (F_2,37.5_ = 1.8, p = 0.18). Successive days did not significantly differ in temperature among control nests (Tukey’s HSD: p>0.80), but experimental nests steadily decreased in temperature over time, such that temperature on day 4 was significantly cooler than on day 1 after implantation (Tukey’s HSD: p = 0.0098; average degrees above ambient on day 1 after implantation: control = 23.0±1.4°C, experimental = 15.6±1.9°C; on day 2: control = 23.1±2.4°C, experimental = 12.8±1.3°C; on day 4: control = 20.5±2.4°C, experimental = 8.6±1.5°C).

## Discussion

Collectively, these results demonstrate that testosterone appears to mediate a behavioral trade-off between parental and aggressive behavior in incubating females: females with experimentally elevated T behaved more aggressively towards a same-sex competitor than did control females, and experimental nests had cooler temperatures, demonstrating that T-implanted females incubated less well than date-matched controls. Prolonged exposure to elevated T had clear fitness costs for females, as there was a robust negative effect of T-implantation on hatching success. These findings likewise demonstrate that females remain behaviorally sensitive to T during the incubation period, contrary to predictions based upon hypotheses used to explain interspecific patterns of behavioral sensitivity/insensitivity to T in males. Elevated T led to the enhancement of aggression at the expense of maternal care in a system where both female parental effort (incubation) and mating effort (defense of a breeding resource against same-sex rivals) are both important to reproductive success during a short breeding season.

The reduction of incubation and enhancement of aggression seen here in female tree swallows is similar to the T-mediated trade-off seen in many species of male birds (see references in Introduction). Because parental care is thought to be particularly important for female reproductive success, selection is thought to favor either lower levels of circulating T in females or reduced sensitivity to T [31], [32]. Consistent with this view, females typically have relatively low levels of circulating T during parental phases of the breeding season [31], [32], and some aspects of maternal care appear to be unresponsive to experimentally elevated T [47], [49]. To my knowledge, however, the results I report here are the first to show that elevated T interferes with female incubation behavior, and this finding demonstrates that female insensitivity to T is not necessarily the norm with respect to incubation behavior, despite the fact that female incubation is clearly essential to female fitness.

Prolonged exposure to T levels at the high end of the natural range of variation for incubating female tree swallows [73] resulted in a robust reduction in incubation and hatching success in this experiment. T-implanted females decreased incubation behavior sufficiently to decrease nest temperatures by approximately 10°C, ultimately leading to nest failure. Plasma T concentrations for female swallows reported here are at the high end of the normal range for tree swallows [73] and other temperate songbirds [31], [32], suggesting that these values reflect natural levels of T (albeit high levels) that a female might experience during incubation. Repeated measurements of incubation temperatures reveal that the behavioral effect of the T-treatment increased over time (i.e. temperature in T-nests became closer and closer to ambient temperature over time), suggesting that the strong effect of T-implantation on incubation was unlikely to be driven by a short-term or supraphysiological surge in T that only affected incubation behavior during the first few days after implantation.

A logical next question is why are females behaviorally sensitive to T if sustained exposure to T levels at the high end of the normal range of variation can have such a detrimental effect on reproductive success. One possibility is that female sensitivity to T may be a byproduct of correlated evolution favoring sensitivity to T in males [74], much as T levels in females may reflect correlated evolution with male T levels [31], [32], [75]. However, previous studies that have compared behavioral sensitivity in males and females of the same species suggest the sexes may be able to evolve somewhat independently, with respect to behavioral sensitivity to T [20], [47], [49], [76]. Another likely possibility is that exposure to the sustained high T levels seen in this experiment is a very rare occurrence, as female T titers typically decline during incubation in most temperate songbirds [31]. Indeed, control females saw a marginal decline in T levels during the study, and control females had significantly lower T levels than experimental females, suggesting that selection for or against sensitivity may not have a chance to operate under unmanipulated circumstances.

A third and not mutually exclusive possibility is that, like males, female patterns of behavioral sensitivity/insensitivity to T also may vary according to the ecology or life history of the species. While sustained and experimentally elevated T was clearly detrimental to hatching success, there are reasons to suspect that the behavioral sensitivity to T seen here may be adaptive under more natural conditions. For example, a short-term T surge may not necessarily have the same strong reduction in egg viability seen in this experimental setting because, like many songbirds, tree swallow eggs are hearty to incubation neglect on a short time scale, e.g. typically one day, but ranging up to several days due to adverse weather conditions [77], [78]. In addition, this study coincided with a 4-day cold snap where ambient temperature = 8.5±0.1°C. Because experimental nests remained much closer to ambient temperatures than controls, it is feasible that this stochastic weather event may have exacerbated the observed negative effect on hatching success. Much more subtle effects on incubation and brooding are known to have deleterious effects on embryo development and ultimately chick condition [58], [79]–[81], and chicks with reduced growth or smaller body mass may be less likely to survive to reproductive age [60], [82]. Indeed, more aggressive female tree swallows tend to have chicks with reduced body mass [53], and the T-mediated trade-off between incubation and aggression observed here provides a mechanism that may partly account for this more subtle cost of aggression observed in nature.

On the whole, the observation that T implants significantly elevated aggression and reduced incubation behavior in female tree swallows is not consistent with the two classic hypotheses for accounting for patterns of behavioral sensitivity/insensitivity in males because these hypotheses would predict that parental care should be unaffected by supplemental T [30], [34]. While these hypotheses were developed to explain patterns of T insensitivity in males, a key question is whether this same logic should apply to a system where T-mediated trade-offs between parental care and aggression are seen in females.

Insights into this mismatch between hypotheses developed for males and the results seen here in females may lie in the assumption (for males) that behavioral sensitivity to T should be favored when T-mediated behaviors are particularly important for reproductive success (for parallelism, I refer to this as the ‘essential mating effort hypothesis’ [4], [10], [27], [36]). Might it be that female tree swallows show behavioral sensitivity to T because this sensitivity is advantageous in the context of same-sex aggression and intrasexual competition? Behavioral sensitivity to T is thought to be beneficial for males in many species because it may enhance a male’s ability to defend a territory or obtain mates [37], [83], though these benefits may very well apply to females as well [46], [84], [85], especially in light of growing evidence that female-female competition for mates and breeding resources may be an essential component of female fitness [41], [43]. Thus, while the sustained elevation of T seen in this study was detrimental to female reproductive success, it may be useful to consider the potential benefits of a short-term, natural elevation in T. Aggressive interactions can lead to a short term rise in T that is thought to prepare individuals for success in future social instability, at least in males of many species [35], [36]. Thus, if a brief spike in T permits an increased aggressive response to the female floaters that intrude at nesting cavities throughout the season, then behavioral sensitivity to T that enables aggressiveness may be adaptive, particularly if a fleeting socially induced elevation in T would have only a fleeting negative effect on incubation behavior. Further sampling of hormones and behavior is needed to address whether the effects of T-implants seen in this study can be extended to natural seasonal or socially-induced changes in T, aggression, or parental care in this system.

Moving forward, explicit consideration of the relative importance of parental care versus aggression will be an important comparison for reconciling patterns of behavioral sensitivity/insensitivity to T in males and in females in other species. For example, the T-induced suppression of paternal care in species with substantial male care (e.g. blue-headed vireo (*Vireo solitarius*) [17]) also does not match the predictions of the essential parental care hypothesis. The essential mating effort hypothesis, however, predicts behavioral sensitivity to T when aggression is particularly important to reproductive success, even if parental behaviors are also underway, as is often the case in males of socially monogamous but genetically promiscuous, biparental species. Shifting gears to females, the links between T and female aggression are rather variable across vertebrates [45], [48], [50], [86]–[88]. In at least two cases, though, T increases female aggression in species where this behavior is associated with significant positive effects on female reproductive success [89], [90], suggesting that the relative benefits of aggression should be contrasted with the importance of parental care to better predict patterns of behavioral sensitivity to T. Currently, a relative scarcity of data on the fitness benefits of female mating effort makes it difficult to test empirically how widely these hypotheses may apply to patterns of T-mediated behavior in females, or whether T-sensitivity would engender the same benefits in females that it would in males. Ultimately, a full understanding of these proximate mechanisms will require the integration of these experimental studies with natural variation in behavioral and hormonal phenotypes in both sexes [6], [87], [90] as well as a metric that can directly compare the relative importance of parental care and aggression.

Many species appear to have resolved the trade-off between mating effort and parental effort with a decrease in T as they shift from mating to parental effort [4], [31], and my finding that endogenous plasma T levels were low in control females is consistent with this view. When the competing demands of parental and mating effort overlap in time, the optimal strategy may also involve compartmentalization of each behavior, e.g. such that aggression depends upon T, but parental care can vary independently of T [11], [66]. Because both parental and aggressive behaviors are often mediated by the same areas of the brain [91] and these nuclei are heavily enervated with sex steroid receptors [92], this potentially adaptive modularity of discrete behaviors may not be possible. Comparative neuroendocrinology demonstrates a range of proximate solutions to this evolutionary problem. Aggression may instead depend upon differences in neural expression of sex steroid receptors or steroidogenic enzymes [93]–[95] or aggression may be mediated by different signaling molecules altogether [13], [96]. Environmentally or socially induced changes in the release of or sensitivity to T [35], [36], [97] or in the amount of free versus bound hormone [29], [98] may likewise permit the adaptive expression of parental and aggressive behaviors. The degree to which each of these proximate mechanisms may underlie the diversity of behavioral responses to T is as of yet unclear across species and sexes [29], [99], and thus awaits further investigation.

## References

[1] Magrath MJL, Komdeur J (2003) Is male care compromised by additional mating opportunity? Trends in Ecology & Evolution 18: 424–430.

[2] Stearns SC (1992) The evolution of life histories. Oxford: Oxford University Press.

[3] Roff DA, Mostowy S, Fairbairn DJ (2002) The evolution of trade-offs: Testing predictions on response to selection and environmental variation. Evolution 56: 84–95. 11913668 10.1111/j.0014-3820.2002.tb00851.x

[4] Wingfield JC, Lynn SE, Soma KK (2001) Avoiding the ‘costs’ of testosterone: Ecological bases of hormone-behavior interactions. Brain Behavior and Evolution 57: 239–251. 10.1159/000047243 11641561

[5] Adkins-Regan E (2005) Hormones and animal social behavior. Princeton, NJ: Princeton University Press.

[6] McGlothlin JW, Jawor JM, Ketterson ED (2007) Natural variation in a testosterone-mediated trade-off between mating effort and parental effort. American Naturalist 170: 864–875. 10.1086/522838 18171169

[7] Folstad I, Karter AJ (1992) Parasites, bright males, and the immunocompetence handicap. American Naturalist 139: 603–622.

[8] Knapp R, Wingfield JC, Bass AH (1999) Steroid hormones and paternal care in the plainfin midshipman fish (*Porichthys notatus*). Hormones and Behavior 35: 81–89. 10049606 10.1006/hbeh.1998.1499

[9] Hirschenhauser K, Oliveira RF (2006) Social modulation of androgens in male vertebrates: meta-analyses of the challenge hypothesis. Animal Behaviour 71: 265–277.

[10] Ketterson ED, Nolan V (1999) Adaptation, exaptation, and constraint: A hormonal perspective. American Naturalist 154: S4–S25. 10.1086/303280 29586711

[11] Hau M (2007) Regulation of male traits by testosterone: implications for the evolution of vertebrate life histories. Bioessays 29: 133–144. 17226801 10.1002/bies.20524

[12] Zera AJ, Harshman LG (2001) The physiology of life history trade-offs in animals. Annual Review of Ecology and Systematics 32: 95–126.

[13] Soma KK, Scotti MAL, Newman AEM, Charlier TD, Demas GE (2008) Novel mechanisms for neuroendocrine regulation of aggression. Frontiers in Neuroendocrinology 29: 476–489. 18280561 10.1016/j.yfrne.2007.12.003

[14] Buntin JD (1996) Neural and hormonal control of parental behavior in birds. In: Rosenblatt JS, Snowdon CT, editors. Advances in the Study of Behavior: Academic Press. 161–213.

[15] De Ridder E, Pinxten R, Eens M (2000) Experimental evidence of a testosterone-induced shift from paternal to mating behaviour in a facultatively polygynous songbird. Behavioral Ecology and Sociobiology 49: 24–30.

[16] Schwagmeyer PL, Schwabl HG, Mock DW (2005) Dynamics of biparental care in house sparrows: hormonal manipulations of paternal contributions. Animal Behaviour 69: 481–488.

[17] Van Roo BL (2004) Exogenous testosterone inhibits several forms of male parental behavior and stimulates song in a monogamous songbird: The blue-headed vireo (*Vireo solitarius*). Hormones and Behavior 46: 678–683. 15555511 10.1016/j.yhbeh.2004.06.011

[18] McDonald PG, Buttemer WA, Astheimer LB (2001) The influence of testosterone on territorial defence and parental behavior in male free-living rufous whistlers, *Pachycephala rufiventris* . Hormones and Behavior 39: 185–194. 11300709 10.1006/hbeh.2001.1644

[19] Lynn SE, Prince LE, Schook DM, Moore IT (2009) Supplementary testosterone inhibits paternal care in a tropically breeding sparrow, *Zonotrichia capensis* . Physiological and Biochemical Zoology 82: 699–708. 19799502 10.1086/605915

[20] Ketterson ED, Nolan V, Wolf L, Ziegenfus C (1992) Testosterone and avian life histories - effects of experimentally elevated testosterone on behavior and correlates of fitness in the dark-eyed junco (*Junco hyemalis*). American Naturalist 140: 980–999.

[21] Silverin B (1980) Effects of long-acting testosterone treatment on free-living pied flycatchers, *Ficedula hypoleuca*, during the breeding period. Animal Behaviour 28: 906–912.

[22] Hegner RE, Wingfield JC (1987) Effects of experimental manipulation of testosterone levels on parental investment and breeding success in male house sparrows. Auk 104: 462–469.

[23] Wingfield JC (1984) Environmental and endocrine control of reproduction in the song sparrow, *Melospiza melodia*. 2. Agonistic interactions as environmental information stimulating secretion of testosterone. General and Comparative Endocrinology 56: 417–424. 6542537 10.1016/0016-6480(84)90084-4

[24] Beletsky LD, Gori DF, Freeman S, Wingfield JC (1995) Testosterone and polygyny in birds. In: Power DM, editor. Current Ornithology. New York, NY: Plenum Press.

[25] Clark MM, Galef BG (1999) A testosterone-mediated trade-off between parental and sexual effort in male mongolian gerbils (*Meriones unguiculatus*). Journal of Comparative Psychology 113: 388–395. 10608562 10.1037/0735-7036.113.4.388

[26] Hunt KE, Hahn TP, Wingfield JC (1999) Endocrine influences on parental care during a short breeding season: testosterone and male parental care in Lapland longspurs (*Calcarius lapponicus*). Behavioral Ecology and Sociobiology 45: 360–369.

[27] Hunt KE, Hahn TP, Wingfield JC (1997) Testosterone implants increase song but not aggression in male Lapland longspurs. Animal Behaviour 54: 1177–1192. 9398371 10.1006/anbe.1997.0558

[28] Trainor BC, Marler CA (2001) Testosterone, paternal behavior, and aggression in the monogamous California mouse (*Peromyscus californicus*). Hormones and Behavior 40: 32–42. 11467882 10.1006/hbeh.2001.1652

[29] Lynn SE (2008) Behavioral insensitivity to testosterone: Why and how does testosterone alter paternal and aggressive behavior in some avian species but not others? General and Comparative Endocrinology 157: 233–240. 18579140 10.1016/j.ygcen.2008.05.009

[30] Lynn SE, Walker BG, Wingfield JC (2005) A phylogenetically controlled test of hypotheses for behavioral insensitivity to testosterone in birds. Hormones and Behavior 47: 170–177. 15664020 10.1016/j.yhbeh.2004.10.004

[31] Ketterson ED, Nolan V, Sandell M (2005) Testosterone in females: Mediator of adaptive traits, constraint on sexual dimorphism, or both? American Naturalist 166: S85–S98. 10.1086/444602 16224714

[32] Møller AP, Garamszegi LZ, Gil D, Hurtrez-Bousses S, Eens M (2005) Correlated evolution of male and female testosterone profiles in birds and its consequences. Behavioral Ecology and Sociobiology 58: 534–544.

[33] Wingfield JC, Hunt KE (2002) Arctic spring: hormone-behavior interactions in a severe environment. Comparative Biochemistry and Physiology B-Biochemistry & Molecular Biology 132: 275–286. 10.1016/s1096-4959(01)00540-1 11997229

[34] Lynn SE, Hayward LS, Benowitz-Fredericks ZM, Wingfield JC (2002) Behavioural insensitivity to supplementary testosterone during the parental phase in the chestnut-collared longspur, *Calcarius ornatus* . Animal Behaviour 63: 795–803.

[35] Goymann W (2009) Social modulation of androgens in male birds. General and Comparative Endocrinology 163: 149–157. 19100740 10.1016/j.ygcen.2008.11.027

[36] Wingfield JC, Hegner RE, Dufty AM, Ball GF (1990) The Challenge Hypothesis - theoretical implications for patterns of testosterone secretion, mating systems, and breeding strategies. American Naturalist 136: 829–846.

[37] Reed WL, Clark ME, Parker PG, Raouf SA, Arguedas N, et al (2006) Physiological effects on demography: A long-term experimental study of testosterone’s effects on fitness. American Naturalist 167: 667–683. 10.1086/503054 16671011

[38] Trivers RL (1972) Parental investment and sexual selection. In: Cambell B, editor. Sexual Selection and the Descent of Man, 1871–1971. London: Heinemann. 136–179.

[39] Shuster SM, Wade MJ (2003) Mating systems and strategies. Princeton, NJ: Princeton University Press.

[40] Clutton-Brock T (2007) Sexual selection in males and females. Science 318: 1882–1885. 18096798 10.1126/science.1133311

[41] Rosvall KA (2011) Intrasexual competition in females: evidence for sexual selection? Behavioral Ecology 22: 1131–1140. 22479137 10.1093/beheco/arr106 PMC3199163

[42] Clutton-Brock T (2009) Sexual selection in females. Animal Behaviour 77: 3–11.

[43] Stockley P, Bro-Jørgensen J (2011) Female competition and its evolutionary consequences in mammals. Biological Reviews of the Cambridge Philosophical Society 86: 341–366. 20636474 10.1111/j.1469-185X.2010.00149.x

[44] Staub NL, DeBeer M (1997) The role of androgens in female vertebrates. General and Comparative Endocrinology 108: 1–24. 9378263 10.1006/gcen.1997.6962

[45] Zysling DA, Greives TJ, Breuner CW, Casto JM, Demas GE, et al (2006) Behavioral and physiological responses to experimentally elevated testosterone in female dark-eyed juncos (*Junco hyemalis carolinensis*). Hormones and Behavior 50: 200–207. 16678179 10.1016/j.yhbeh.2006.03.004

[46] Veiga JP, Polo V (2008) Fitness consequences of increased testosterone levels in female spotless starlings. American Naturalist 172: 42–53. 10.1086/587850 18532881

[47] O’Neal DM, Reichard DG, Pavilis K, Ketterson ED (2008) Experimentally-elevated testosterone, female parental care, and reproductive success in a songbird, the Dark-eyed junco (*Junco hyemalis*). Hormones and Behavior 54: 571–578. 18585386 10.1016/j.yhbeh.2008.05.017

[48] Sandell MI (2007) Exogenous testosterone increases female aggression in the European starling (*Sturnus vulgaris*). Behavioral Ecology and Sociobiology 62: 255–262.

[49] Clotfelter ED, O’Neal DM, Gaudioso JM, Casto JM, Parker-Renga IM, et al (2004) Consequences of elevating plasma testosterone in females of a socially monogamous songbird: evidence of constraints on male evolution? Hormones and Behavior 46: 171–178. 15256307 10.1016/j.yhbeh.2004.03.003

[50] De Ridder E, Pinxten R, Mees V, Eens M (2002) Short- and long-term effects of male-like concentrations of testosterone on female European starlings (*Sturnus vulgaris*). Auk 119: 487–497.

[51] Kriner E, Schwabl H (1991) Control of winter song and territorial aggression of female robins (*Erithacus rubecula*) by testosterone. Ethology 87: 37–44. 10.1016/0018-506x(91)90049-n 2066079

[52] Rosvall KA (2010) Do males offset the cost of female aggression? An experimental test in a biparental songbird. Behavioral Ecology 21: 161–168.

[53] Rosvall KA (2011) Cost of female intrasexual aggression in terms of offspring quality: a cross-fostering study. Ethology 117: 1–13.

[54] Robertson RJ, Stutchbury BJ, Cohen RR (1992) Tree swallow (*Tachycineta bicolor*). The Birds of North America, No 11: Academy of Natural Sciences, Philadelphia, PA and American Ornithologists’ Union, Washington, D. C: 1–26.

[55] Hunt K, Wingfield JC, Astheimer LB, Buttemer WA, Hahn TP (1995) Temporal patterns of territorial behavior and circulating testosterone in the Lapland longspur and other arctic Passerines. American Zoologist 35: 274–284.

[56] Lynn SE, Wingfield JC (2008) Dissociation of testosterone and aggressive behavior during the breeding season in male chestnut-collared longspurs, *Calcarius ornatus* . General and Comparative Endocrinology 156: 181–189. 18275961 10.1016/j.ygcen.2008.01.004

[57] Poole A, editor (2005) The Birds of North America. Ithaca, NY: Cornell Laboratory of Ornithology.

[58] Ardia DR, Perez JH, Clotfelter ED (2010) Experimental cooling during incubation leads to reduced innate immunity and body condition in nestling tree swallows. Proceedings of the Royal Society B: Biological Sciences 277: 1881–1888. 20147326 10.1098/rspb.2009.2138 PMC2871872

[59] Lombardo MP, Bosman RM, Faro CA, Houtteman SG, Kluisza TS (1995) Effect of feathers as nest insulation on incubation behavior and reproductive performance of tree swallows (*Tachycineta bicolor*). Auk 112: 973–981.

[60] McCarty JP (2001) Variation in growth of nestling tree swallows across multiple temporal and spatial scales. Auk 118: 176–190.

[61] Stutchbury BJ, Robertson RJ (1985) Floating populations of female tree swallows. Auk 102: 651–654.

[62] Leffelaar D, Robertson RJ (1985) Nest usurpation and female competition for breeding opportunities by tree swallows. Wilson Bulletin 97: 221–224.

[63] Stutchbury BJ, Robertson RJ (1987) Behavioral tactics of subadult female floaters in the tree swallow. Behavioral Ecology and Sociobiology 20: 413–419.

[64] Rosvall KA (2008) Sexual selection on aggressiveness in females: evidence from an experimental test with tree swallows. Animal Behaviour 75: 1603–1610.

[65] Stutchbury BJ, Robertson RJ (1987) Signaling subordinate and female status - two hypotheses for the adaptive significance of subadult plumage in female tree swallows. Auk 104: 717–723.

[66] Ketterson ED, Atwell JW, McGlothlin JW (2009) Phenotypic integration and independence: Hormones, performance, and response to environmental change. Integrative and Comparative Biology 49: 365–379. 21665827 10.1093/icb/icp057 PMC4012227

[67] Dunn PO, Robertson RJ, Michaudfreeman D, Boag PT (1994) Extra-pair paternity in tree swallows - Why do females mate with more than one male. Behavioral Ecology and Sociobiology 35: 273–281.

[68] Desteven D (1978) Influence of age on breeding biology of tree swallow *Iridoprocne bicolor* . Ibis 120: 516–523.

[69] Ardia DR, Clotfelter ED (2007) Individual quality and age affect responses to an energetic constraint in a cavity-nesting bird. Behavioral Ecology 18: 259–266.

[70] Stutchbury BJ, Robertson RJ (1988) Within-season and age-related patterns of reproductive-performance in female tree swallows (*Tachycineta bicolor*). Canadian Journal of Zoology 66: 827–834.

[71] Hussell DJT (1983) Age and plumage color in female tree swallows. Journal of Field Ornithology 54: 312–318.

[72] Fusani L (2008) Endocrinology in field studies: Problems and solutions for the experimental design. General and Comparative Endocrinology 157: 249–253. 18550056 10.1016/j.ygcen.2008.04.016

[73] Bishop CA, Van Der Kraak GJ, Ng P, Smits JEG, Hontela A (1998) Health of tree swallows (*Tachycineta bicolor*) nesting in pesticide-sprayed apple orchards in Ontario, Canada. II. Sex and thyroid hormone concentrations and testes development. Journal of Toxicology and Environmental Health-Part A 55: 561–581. 9885998 10.1080/009841098158250

[74] Lande R, Arnold SJ (1983) The measurement of selection on correlated characters. Evolution 37: 1210–1226. 28556011 10.1111/j.1558-5646.1983.tb00236.x

[75] Mank JE (2007) The evolution of sexually selected traits and antagonistic androgen expression in actinopterygiian fishes. American Naturalist 169: 142–149. 10.1086/510103 17206593

[76] Schoech SJ, Ketterson ED, Nolan V, Sharp PJ, Buntin JD (1998) The effect of exogenous testosterone on parental behavior, plasma prolactin, and prolactin binding sites in dark-eyed juncos. Hormones and Behavior 34: 1–10. 9735223 10.1006/hbeh.1998.1455

[77] Wang JM, Beissinger SR (2009) Variation in the onset of incubation and its influence on avian hatching success and asynchrony. Animal Behaviour 78: 601–613.

[78] Wang JM, Beissinger SR (2011) Partial incubation in birds: its occurrence, function, and quantification. Auk 128: 454–466.

[79] Cresswell W, Holt S, Reid JM, Whitfield DP, Mellanby RJ, et al (2004) The energetic costs of egg heating constrain incubation attendance but do not determine daily energy expenditure in the pectoral sandpiper. Behavioral Ecology 15: 498–507.

[80] Hepp GR, Kennamer RA, Johnson MH (2006) Maternal effects in Wood Ducks: incubation temperature influences incubation period and neonate phenotype. Functional Ecology 20: 307–314.

[81] Perez JH, Ardia DR, Chad EK, Clotfelter ED (2008) Experimental heating reveals nest temperature affects nestling condition in tree swallows (*Tachycineta bicolor*). Biology Letters 4: 468–471. 18628112 10.1098/rsbl.2008.0266 PMC2610083

[82] Gebhardt-Henrich S, Richner H (1998) Causes of growth variation and its consequences for fitness. In: Starck JM, Ricklefs RE, editors. Avian growth and development: evolution within the altricial-precocial spectrum. Oxford: Oxford University Press. 324–339.

[83] Raouf SA, Parker PG, Ketterson ED, Nolan V, Ziegenfus C (1997) Testosterone affects reproductive success by influencing extra-pair fertilizations in male dark-eyed juncos (Aves: *Junco hyemalis*). Proceedings of the Royal Society B: Biological Sciences 264: 1599–1603.

[84] Langmore NE, Davies NB (1997) Female dunnocks use vocalizations to compete for males. Animal Behaviour 53: 881–890.

[85] Eens M, Pinxten R (2000) Sex-role reversal in vertebrates: behavioural and endocrinological accounts. Behavioural Processes 51: 135–147. 11074317 10.1016/s0376-6357(00)00124-8

[86] Elekonich MM, Wingfield JC (2000) Seasonality and hormonal control of territorial aggression in female song sparrows (Passeriformes: Emberizidae: *Melospiza melodia*). Ethology 106: 493–510.

[87] Jawor JM, Young R, Ketterson ED (2006) Females competing to reproduce: Dominance matters but testosterone may not. Hormones and Behavior 49: 362–368. 16226754 10.1016/j.yhbeh.2005.08.009

[88] While GM, Isaksson C, McEvoy J, Sinn DL, Komdeur J, et al (2010) Repeatable intra-individual variation in plasma testosterone concentration and its sex-specific link to aggression in a social lizard. Hormones and Behavior 58: 208–213. 20361965 10.1016/j.yhbeh.2010.03.016

[89] Sandell MI (1998) Female aggression and the maintenance of monogamy: female behaviour predicts male mating status in European starlings. Proceedings of the Royal Society B: Biological Sciences 265: 1307–1311.

[90] Cain KE, Ketterson ED (2012) Competitive females are successful females; phenotype, mechanism, and selection in a common songbird. Behavioral Ecology and Sociobiology 66: 241–252. 22345899 10.1007/s00265-011-1272-5 PMC3278083

[91] Goodson JL (2005) The vertebrate social behavior network: Evolutionary themes and variations. Hormones and Behavior 48: 11–22. 15885690 10.1016/j.yhbeh.2005.02.003 PMC2570781

[92] Gahr M, Metzdorf R (1997) Distribution and dynamics in the expression of androgen and estrogen receptors in vocal control systems of songbirds. Brain Research Bulletin 44: 509–517. 9370218 10.1016/s0361-9230(97)00233-5

[93] Canoine V, Fusani L, Schlinger B, Hau M (2007) Low sex steroids, high steroid receptors: Increasing the sensitivity of the nonreproductive brain. Developmental Neurobiology 67: 57–67. 17443772 10.1002/dneu.20296

[94] Voigt C, Goymann W (2007) Sex-role reversal is reflected in the brain of African black coucals (*Centropus grillii*). Developmental Neurobiology 67: 1560–1573. 17542014 10.1002/dneu.20528

[95] Rosvall KA, Bergeon Burns CM, Barske J, Goodson JL, Schlinger BA, et al (2012) Neural sensitivity to sex steroids predicts individual differences in aggression: implications for behavioural evolution. Proceedings of the Royal Society B: Biological Sciences 279: 3547–3555. 22673360 10.1098/rspb.2012.0442 PMC3396890

[96] Goymann W, Wittenzellner A, Schwabl I, Makomba M (2008) Progesterone modulates aggression in sex-role reversed female African black coucals. Proceedings of the Royal Society B: Biological Sciences 275: 1053–1060. 18252672 10.1098/rspb.2007.1707 PMC2600909

[97] Oliveira RF (2009) Social behavior in context: Hormonal modulation of behavioral plasticity and social competence. Integrative and Comparative Biology 49: 423–440. 21665831 10.1093/icb/icp055

[98] Swett MB, Breuner CW (2008) Interaction of testosterone, corticosterone and corticosterone binding globulin in the white-throated sparrow (*Zonotrichia albicollis*). Comparative Biochemistry and Physiology A-Molecular & Integrative Physiology 151: 226–231. 10.1016/j.cbpa.2008.06.031 18644248

[99] Ball GF, Balthazart J (2008) Individual variation and the endocrine regulation of behaviour and physiology in birds: a cellular/molecular perspective. Philosophical Transactions of the Royal Society B: Biological Sciences 363: 1699–1710. 10.1098/rstb.2007.0010 PMC2606728 18048288

